# Towards a systematic framework to assess restoration success of interventions in coral reef ecosystems

**DOI:** 10.1371/journal.pone.0331083

**Published:** 2026-03-09

**Authors:** Aldo Croquer, Sergio D. Guendulain-García, Jonathan S. Lefcheck, Leah Harper, Elizabeth Shaver, Rita I. Sellares-Blasco, Maria F. Villapando, Ainhoa L. Zubillaga, Rebecca Garcia-Camps, Andreina Rivera, Eva Salas, Jennifer Humberstone, Lorenzo Alvarez-Filip

**Affiliations:** 1 The Nature Conservancy, Caribbean Division, Marine Conservation Program, Central Caribbean,; 2 Unidad Académica de Sistemas Arrecifales del ICML, Universidad Nacional Autónoma de México (UNAM), Puerto Morelos, Quintana Roo, México; 3 University of Maryland Center for Environmental Science, Cambridge, Maryland, United States of America; 4 Tennenbaum Marine Observatories Network, MarineGEO, Smithsonian Environmental Research Center, Edgewater, Maryland United States of America; 5 Fundación Dominicana de Estudios Marinos, Bayahibe, La Altagracia, Dominican Republic; 6 Marine Innovation Center, Fundación Punta Cana, Punta Cana, La Altagracia, Dominican Republic; 7 The Nature Conservancy, Sacramento, California, United States of America; University of the Ryukyus, JAPAN

## Abstract

An ecosystem is defined as a collection of organisms that move energy within and outside of a system, while sustaining both the system itself and the multiple services that benefit humanity. Ecosystem restoration, then, is ultimately concerned with reviving and maintaining ecosystem processes by repopulating organisms and enhancing the habitat after periods of disturbance or loss. Whether interventions are considered “successful” depends on three criteria: 1) were the goals/outcomes clearly defined before implementing the intervention; 2) did the outcome arise directly from the intervention, and 3) does the outcome reflect a functioning ecosystem in the long term (e.g., > 10 years)? The answers to these questions have been challenging for coral restoration practitioners, as they are often hindered by the lack of predefined hypotheses and rigorous experimental design and by confusion between metrics quantifying coral production and outplanting efforts rather than recovery of community structure and ecosystem functioning. As a result, the impacts of restoration efforts are inconsistently and often incorrectly interpreted, and funding is often tied to intervention activities instead of outcomes. Here, we present a framework to implementing robust experimental designs and measure more relevant ecosystem indicators in order to assess the impacts of interventions and promote more informed and effective restoration outcomes. We then illustrate these concepts by reviewing coral restoration-specific case studies to demonstrate the degree to which successful outcomes under such a framework have been achieved. Through these practical recommendations, we hope to support coral restoration practitioners in designing and executing future interventions, and to encourage the broader community, including funders, to adopt a more systematic framework to evaluate and report restoration success.

## Introduction

Coral reefs in the Anthropocene have been declining at unprecedented rates [1,2]. The combination of local and global stressors and the extirpation of foundation and keystone species have had negative impacts on reef functioning [3,4], reducing the natural potential of these ecosystems to recover from disturbance [5,6] and their capacity to deliver goods and services to millions of people [7,8]. As coral reefs continue to decline and their ecological resilience is compromised in an era dominated by climate change, many restoration practices have emerged with the ambitious intention of halting, mitigating, and ultimately reversing the local loss of coral reefs in nature [9].

The field of coral restoration has made significant recent advances, with most efforts being focused on developing and refining breeding and outplanting methods and advancing technologies to scale-up coral production/survivorship at lower costs [9–12]. In contrast, conceptual frameworks involving experimental design, community ecology, ecosystem ecology, and landscape dynamics have received much less attention by the coral restoration community [13], as has been the case with ecological restoration generally [14]. Furthermore, the impacts of restorations have failed to match the extent of coral ecosystem loss (i.e., hundreds of hectares) over meaningful time frames (i.e., > 10 years) [15].

Intact coral reef ecosystems are defined by a complex habitat supporting diverse assemblages of organisms that sustain a range of biogeochemical (e.g., carbonate accretion, nutrient cycling) and ecological processes (e.g., herbivory, predation) [16]. Despite recent achievements in coral restoration interventions targeting larger scales (e.g., tens of hectares) and more efficient coral propagation methods and projects, the majority of efforts measure success on colony or even fragment-level attributes, such as number of outplants and/or their survival over time [9]. Only a few published coral restoration studies assess ecosystem-wide attributes such as structural complexity or enhanced biodiversity [17–19]. Compounding this issue, most coral restoration projects lack proper experimental design, appropriate replication, or fail to systematically follow the change of ecosystemic properties over time. Therefore, the essential ecological question of how coral restoration success is measured, and how quantitative experimental evidence can be used to demonstrate improvement and/or recovery of former ecosystem structure and function, remains unanswered in most coral restoration programs [13,15,20]. One of the fundamental challenges for the field of coral reef restoration is how practitioners can effectively assess and report on the success or failure of their projects to ensure valuable resources are not wasted in scaling ineffective strategies and future projects can be adapted to emerging needs and threats.

Whether interventions are “successful” requires the consideration of three major criteria: 1) were the goals/outcomes clearly defined before implementing the intervention; 2) were the goals met and did the outcome arise because of the intervention; and 3) does the outcome reflect a functioning ecosystem, such that actual ecological restoration is being achieved in the long term (e.g., > 10 years)? Here, we present a conceptual and experimental framework to guide practitioners to answer these questions more successfully. We discuss the need for experimental frameworks in coral restoration projects so that results can be quantitatively measured and argue for the use of monitoring metrics focused on ecosystem structure and functioning rather than restoration effort and/or colony performance (e.g., production and outplanting of corals, outplant survivorship and outplant growth rates). We then provide specific examples of how conceptual and experimental frameworks have been used in coral restoration projects.

### Ecological restoration conceptual framework

Ecological restoration aims to assist the recovery of a degraded, damaged or destroyed ecosystem, generally (but not always) in the direction of a historical reference [21,22]. The starting point of any restoration intervention is always a degraded or disturbed ecosystem with limited capacity for natural recovery. Any attempt of ecological restoration must acknowledge the importance of natural recruitment and ecological succession and how species can or cannot reassemble and change over time following natural and/or anthropogenic disturbance [23–26]. Differentiating between the natural and intervention paths to restoration, and at what level of ecological organization is being considered, is vital to set the scope and goals of any restoration program [21,27–29].

Often, it is virtually impossible to achieve full recovery either because of the lack of a historical reference, drastic changes in prevailing environmental conditions (such as temperature), and/or because “restoration as the outcome” has sometimes become synonymous with “intervention as the process of rearing and outplanting corals” [30]. Even when baselines are available, they only represent a snapshot of a highly dynamic and complex ecosystem. Because returning to historic baselines is likely not feasible in the face of climate change and that, in many ways, we don’t know what the new “baseline” for functioning, resilient reefs are, reaching recovery only means the system returned to the point we set as “desirable” according to expert criteria and perceptions which change over time (i.e., the shifting base line syndrome) [31]. In addition, baselines and “desirable functioning” might be strongly site-dependent (e.g., enhancing habitat vs. enhancing climate resilience). In such cases, interventions must aim to rehabilitate, enhance or “improve” community structure and ecosystem function rather than restore [21,22,27]. Here, we regard reef restoration as a compilation of human interventions implemented to aid the recovery (e.g., restore, enhance, rehabilitate) or even replace a specific set of collective and emergent properties of a coral community and/or a coral reef ecosystem [10]. Therefore, having a clear idea of emergent properties that define community structure and ecosystem functionality is essential for coral restoration practitioners.

**Fig 1:**
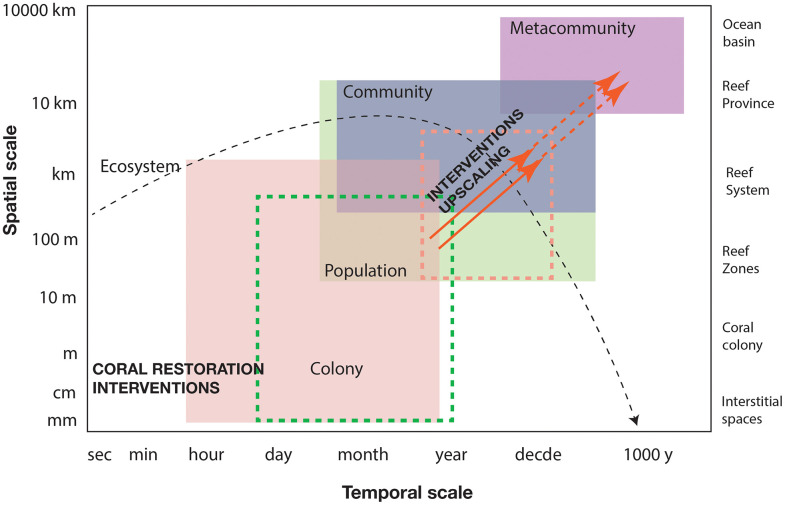
Schematic representation of levels of organization, spatial and temporal scales that can be impacted by coral restoration efforts. Solid arrows represent expected outcomes that can be upscaled. Dashed arrows represent outcomes that require modelling exercises to prove expected success. Green dash box encapsules the levels of organization and temporal and spatial scales where benefits of restoration projects have been reported.

Likely, most interventions currently implemented by coral restoration practitioners are benefiting organisms and populations within hectares in the short-middle term (<10 years, Fig 1). Restoration of species associated with physical structures provided by corals have their own specific functions and interactions that must also be considered. Corals are a diverse group of organisms with different structural and functional characteristics [2,3,32,33] that have various implications for their use as habitat and for their capacity to provide additional services (such as wave attenuation and shoreline protection) [34]. Therefore, measuring success in coral restoration should not only be reflected in the increase in the number of corals writ large, but rather in the desirable attributes of different coral species that maximize the functionality of the ecosystem (i.e., “functional diversity”). Such positive impacts for upper levels of organization (e.g., metacommunities and ecosystems), across reef systems and provinces have not yet been demonstrated and might require complex modeling exercises to demonstrate positive outcomes in the long term (Fig 1). Thus, a functional approach for coral restoration is one that recognizes both the foundational structure as well as the emergent biodiversity and functions associated with coral ecosystems [35,36]. What matters is not only the taxonomic identity of reef organisms, but the specific ecological role or impact that species has on the ecosystem [16,37]. Restoring, rehabilitating, or enhancing ecosystem structure and function is not trivial and requires a formal adoption of ecosystem functioning monitoring metrics as part of a robust experimental design that will allow practitioners to assess the outcomes of interventions.

### Experimental design conceptual framework

The role of experimental ecology in coral restoration has long been acknowledged [9,20,38] but rarely operationalized. Recently, coral restoration experiments have being described as discrete “fast fail” activities designed to develop techniques and detect problems as early as is feasible in the development phase and as pilot studies [39], but not as full-fledged frameworks to follow and evaluate restorations over the timescales it takes to recovery ecosystem structure and ecological processes. Furthermore, it has been argued that restoration ecology experiments must be separated from collective assessments of coral restoration activity [39]. However, restoration activities must always be designed to quantify the effect size [40,41] of cumulative interventions and to separate these long-term effects from other natural/uncontrolled sources of variation in the experiment (e.g., natural recovery). Otherwise, it is impossible to ascribe observed outcomes directly to the intervention, muddying our understanding of why and how restorations succeed (or do not) and leading to future failures. Thus, any human intervention aimed at reassembling the natural complexity of a coral community or a coral reef ecosystem constitutes-by definition- a multiscale long-term manipulative field experiment [42–44].

**Fig 2:**
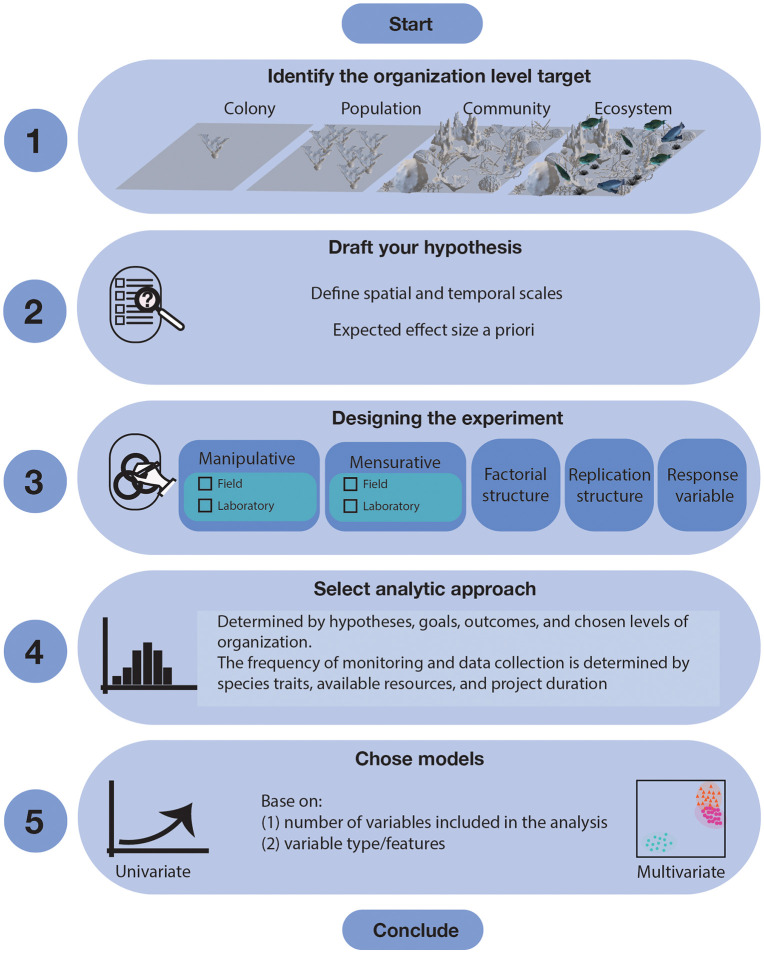
Schematic representation of key steps in the design of a coral restoration project targeting community and ecosystems levels of organization.

A long-term manipulative experiment should have a clearly articulated hypothesis (expected outcome arising from the intervention) based on the level of organization being targeted (species/community/ecosystem), as well as a set of independent factors and dependent (response) variables that must be properly selected to measure the outcome of the experiment over the chosen spatial and temporal scales [44–48]. Principles of proper randomization, spatial array of treatments and replication of experimental and operation units are all essential to avoid pseudoreplication and for proper statistical inference and interpretation of results [47,48]. Finally, because resources for restoration are limited, we recommend following the hypothetical-deductive reasoning whereby hypotheses are drafted *before* data is collected and the hypotheses are falsifiable [46] (Fig 2); rather than the opposite inductive-Baconian approach in which data is collected first to draft and confirm hypotheses [46].

Mensurative and manipulative experiments can be conducted both in the field and in the laboratory. Active restoration based on coral outplanting is a good example of a manipulative experiment, whereas passive restoration based on mitigation constitutes an example of a mensurative experiment. The factorial structure includes a series of decisions regarding number and type of factors and how they related to each other. Replication structure includes a clear differentiation between experimental and operational units, proper randomization and independence to avoid confounding responses. Response variables are measured upon operation units belonging to a target biological population. The selection of variables and the frequency of monitoring will be determined by the duration of the experiment, available resources, established capacity and the life-history traits of species used in the experiment.

### Hypothesis construction for a coral restoration experiment

A clear hypothesis being tested is fundamental for any experiment [46]. In a restoration experiment, the underlying hypothesis encompasses predictions about specific outcomes of human interventions aimed to restore (i.e., bring back to the original condition), rehabilitate (i.e., recover, enhance, or “improve”), and/or replace the community structure and function of an ecosystem in a particular area within a specific period. When hypotheses are designed and tested using a frequentist approach, an effect size must be set *a priori* so that the experimenter predicts the magnitude of the expected change that interventions will produce in the target area within a period (e.g., a 5% increase in coral cover or colony density) [46] (Fig 2). Effect size should always be proportional to outplanting effort, established capacity and reasonable/justifiable cost-effective interventions so that all targets are timebound for every project. Furthermore, expectations should be set based on the current status of the reef and what can reasonably be achieved given knowledge of past interventions or pilot studies. A clear hypothesis or prediction would be that a series of interventions (e.g., outplanting of target coral species) will improve the habitats (e.g., increase coral cover and abundance) and concomitantly a “portion” of ecosystem functionality within a site for a known period relative to a reference (Fig 2). This process is similar and can be useful for developing SMART (Specific, Measurable, Achievable, Relevant and Time-bound) goals for coral restoration project planning and design [29].

**Fig 3:**
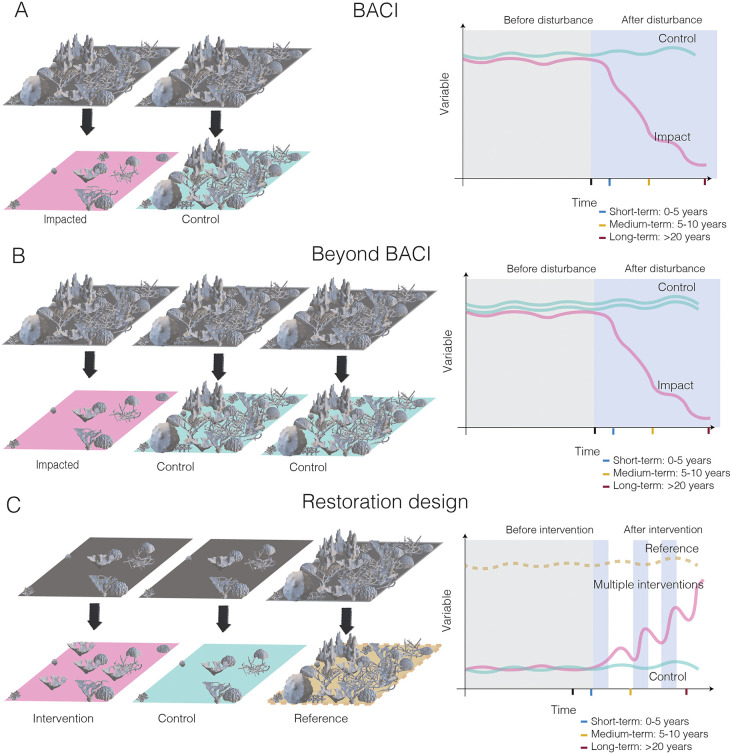
Fundamental differences between: (a) BACI, (b) Beyond BACI and (c) Restoration experimental designs in terms of levels of treatments. The theoretical expected outcomes from the different designs are shown in the right plots; before (light gray) and after (light blue) disturbances and interventions. Time scales are provided in year intervals.

Restoration experiments are often wrongly regarded as Before-After-Control Impact (BACI) and Beyond BACI designs under the assumption that interventions are human activities with positive impacts. However, there are striking differences between these designs (Fig 3). First, a BACI design compares two sites that are different in ecological status: (1) disturbed and (2) undisturbed [49,50]. The Beyond BACI approach replicates more than one control site leading to asymmetric designs because normally the impacted site cannot be replicated [49]. In contrast, a restoration design compares three sites: (1) a disturbed/degraded area that will not benefit from interventions (i.e., the “control” site), (2) a similar area where the interventions are deployed (i.e., the “intervention” site), and (3) an area that reflects the expected outcome of interventions (i.e., the “reference” site) that is in “better” condition compared to control and intervention sites, but not necessarily pristine [21,22,51]. However, the control and intervention sites may still be disturbed due to overarching factors, so any improvement from the intervention must be measured relative to such changes over time. The null hypothesis for a restoration experiment is that interventions will not “improve” or have a positive effect on any emergent property of the chosen level of organization. Note that time (e.g., months, years or decades) and space (e.g., m^2^, hectares, km^2^) are important concepts to consider (Fig 3) but should not change the way the hypothesis is drafted nor the fact that restoration is a field manipulative experiment.

Finally, the meaning of a reference site deserves special attention. A reference site could be a pristine area (if any), historical data when available or a site that is in better condition and resembles what the restoration project strives to achieve [51]. If the objective of the intervention is to replace the ecosystem because it is impossible to bring back the ecosystem as it was, then the reference site must portray what we want to achieve post interventions. Thus, whether the reference site is located on the same reef where interventions are implemented is not as relevant as knowing the desired outcome of these interventions. The take home message is that every restoration project should have a reference for the desired outcome of the interventions, and without a reference it is impossible to ascertain what has been restored in relation to a desirable ecosystem state [21,51].

In a restoration experiment, we aim not only to detect the dissimilarity between the control and the intervention site but also to determine how similar the intervention site becomes in relation to the reference site after restoration interventions are implemented [51]. Thus, the control site in a BACI is not the same as the control for a restoration design [51]; rather, it is equivalent to the reference site [51]. Ultimately, interventions made in a restoration experiment must not be regarded as a disturbance because BACI “impact” sites and restoration “intervention” sites are generally expected to have opposite temporal trajectories (i.e., declining for the first and “improving” for the latter) (Fig 3). Therefore, the ecological prediction of a successful restoration experiment should be that, over time, intervention sites will become more dissimilar compared to control and concomitantly more similar to the reference site [51]. However, this may not always be the case: for example, a loss of 5% cover at the intervention site may still be considered successful if there is a corresponding loss of 10% cover at the control or especially the reference site (i.e., the outcome is beyond what would be expected in the absence of intervention).

This design has additional causal implications for assessing restoration outcomes. Another way of phrasing the hypothesis is what would have happened if the system had *not* intervened, or was the intervention necessary? This counterfactual is never capable of being observed, since the investigator has changed the system simply through the act of the intervention. Thus, a suitable counterfactual must be developed and defended to reflect the state of the system in the absence of intervention [14]. This can generally be achieved through the careful selection and implementation of the control site to capture the same environmental and ecological conditions as the restoration site to generate a fair comparison [52], and in setting appropriate targets using the reference site. Consider the scenario in which a restoration site achieves higher coral cover after the intervention. In the absence of any additional context, it would be tempting to attribute the successful outcome to the restoration interventions. However, imagine that the control site also increased in coral cover by means of natural recovery. With this new information, alternative hypotheses could be developed and tested: for example, a large-scale recruitment event has led to region-wide recovery that would have regardless of the intervention [53,54]. It may even be that the control site increased in coral cover beyond that of the restoration site, suggesting that the intervention may have had a negative impact despite an overall increase in hard coral cover (for example, through disturbance of the benthos by restoration activities). Nevertheless, if this level is still below that found at the reference site, the results may suggest further intervention is needed even in the presence of natural recovery. Without establishing proper control and reference sites reflective of pre-intervention conditions and large-scale trajectories under the above design framework, a causal test of the null hypothesis is simply not possible.

Additionally, there are critical, but often not perceived, differences between BACI and restoration designs regarding the relative importance of committing type I (alpha) or Type II (beta) inference errors. For a BACI design, it is more critical to avoid type II errors, or the incorrect retention of the null hypothesis of lack of impacts. For a restoration experiment, avoiding Type I error, or an incorrect rejection of the true null hypothesis of lack of restoration success. In other words, is it better to mistakenly reject a successful outcome (Type II error) than to wrongly declare a failed outcome as a success (Type I error). Arguably, the latter scenario would lead to the most damage, as not only has the restoration failed to achieve the expected outcome, but the wrongful perception of success may lead restorationists to repeat these failed practices into the future, propagating failure to other restorations.

### Factorial design to test intervention success

Once the null hypothesis is clearly formulated and the errors of inference are established *a priori*, the next step is to conceptualize a factorial design in the experiment allowing to test the null hypothesis of no intervention success [46]. Factors can be fixed or random and orthogonal or nested. These concepts will not be addressed here as they are clearly explained elsewhere [46] but deciding based on the hypothesis to be tested is extremely relevant since they have a direct impact on how the statistics are calculated in the models and in the interpretation of results [46]. The term replicate is rather a qualifier as in replicated experiments, experimental units, blocks, operation (evaluation) units, samples and subsamples or any other unit of analysis [48]. Misinterpretation of the units of analysis and/or lack of independence among these units will lead to simple, temporal, sacrificial, and/or test-qualified sacrificial pseudoreplication [47,48,55].

While a truly comprehensive discussion of full suite of analytical tools available to restoration practitioners is beyond this article, we provide some general guidelines for drawing statistical inference from our proposed design. In terms of univariate responses, such as changes in percent cover or species richness, we recommend a regression-based approach with the following key fixed effects of interest: (1) treatment (restoration, control, and reference); (2) time (either before or after the intervention, or the number of days since the initiation in the event of repeated observations); and (3) a treatment-by-time interaction, to test whether any treatment effects are emerging before vs. after the intervention or over the course of post-restoration monitoring. It is important to note that the plots represent the experimental units in which the treatments are replicated. If the hypothesis involves predictions encompassing broader spatial scales (e.g., multiple locations), then sites must also be replicated (Supplementary Figure S1 in S1 Supplementary). The use of post-hoc contrasts can identify which treatments differ in the event of a significant treatment effect, which is key in distinguishing if, say, the intervention site differs from the baseline, or whether the baseline differs from the reference. Additional covariates that may affect the outcomes—such as depth, environment (e.g., temperature), and design factors such as effort (number of outplants)—may be additionally modeled as fixed effects.

It is also important to account for sources of known but uncontrolled variation, such as repeated observations within the same plot (subsamples or pseudoreplication) or multiple plots within and/or among sites (a nested or hierarchical sampling design). The most straightforward implementation is to specify these known sources as so-called “random effects” in a generalized linear mixed effects modeling framework—this approach not only prevents them from being included in the error (unexplained) variance but allows for less biased (and often more precise) estimates of the fixed effects. If the practitioner suspects that the treatment effects may vary across multiple sites, for example, if one site is more degraded and thus may respond more slowly or not at all to the intervention, then it may be valuable to further specify a random slope of treatment. Further discussion of specifying fixed and random effects can be found in [56].

We note that such mixed-effects models are analogous to specifying a nested structure in classic Analysis of Variance (ANOVA) procedures, which may be familiar to many practitioners due to their approachability and ease of estimation. Unlike ANOVA, which partitions variance using sums-of-squares estimation, modern linear modeling generally uses Maximum Likelihood, which is ideal for unbalanced or non-orthogonal designs (for example when replicates are lost) or when the response is highly skewed (such as for abundance or density), are only positive integers (species richness), or binary (such as presence-absence). While both are valid, they may require subtly different specifications to meet the statistical assumptions of the two tests and accommodate the underlying data-generating process.

If the restoration occurred over multiple locations and there is an specific hypothesis about sites, we additionally suggest to include a fixed effect of site, assuming this term is not conflated with treatment (i.e., each site represents a different treatment), for two reasons: first, it is often of interest to understand whether restorations perform differently at different sites, and second, this term can account for omitted variable bias (OBV), or the hidden influence of unmeasured factors that may lead to spurious conclusions. A discussion of the use of fixed effects (and other approaches) to reduce OBV can be found in [57]. Alternatively, sites could be modelled as random factor if the restoration practitioner is interested in exploring if intervention outcomes are scalable or variable or can be extrapolated to other sites.

Restoration experiments could be planned to be even more complex, adding depths, habitats, localities or regions and any kind of spatial and temporal variation.. Furthermore, when interventions involve the introduction of artifacts (e.g., exclusion nets, artificial substrates, frames, devices for shading, media to deliver terapeuthic treatments) or any kind of device that might influence the response variable, procedural controls for these artifacts are essential to validate results [46]. The important take-home message for coral restoration practitioners is that the adoption of an experimental framework opens the chance to choose for proper statistical parametric (e.g., ANOVA and GLM) and semiparametric models based on permutations and similarity/distance matrices (e.g., PERMANOVA) that allow the separation of the true effect of their interventions from a series of uncontrolled factors and relevant interactions.

### Response variables: univariate versus multivariate approach

A restoration experiment may focus on measuring a single variable (i.e., univariate approach) and/or measuring multiple response variables (i.e., multivariate approach). The univariate approach is often easier to interpret but it misses relevant information when dealing with complex and functionally diverse ecosystems. On the other hand, the multivariate approach is more difficult to interpret, but it captures the actual complexity of the system which is more in line with the goals of ecological restoration at the community and ecosystem levels of organization.

Ideally, a coral restoration program should collect data on different assemblages and functional traits (e.g., benthic species, fish, invertebrates, habitat complexity, herbivory, predation rates, etc.) to test if interventions are improving and/or recovering the structure and function of the ecosystem. The goal is not only to show recovery of hard coral cover, for example, but also to be able to link interventions with “improvement” of habitat structural complexity and concomitantly the development of communities that actively interact with that habitat over space and time. Thus, considering spatial and temporal dynamics across different scales is crucial for assessing recovery of emergent properties at community and/or ecosystems levels of organization.

Emergent properties are unique and arise through the integration of processes up the levels of organization (genetic/organismal, population, community, and ecosystems) [58]. Levels of organization occur across boundaries, including spatial (from millimeters to hundreds of thousands of km) and temporal (from minutes to hundreds to thousands of years) scales [58].Thus, choosing the proper metrics to assess coral restoration success is not trivial as it involves a clear understanding of the target level of organization, the interconnection of oceanographic, geological and ecological processes, and relevant spatial and temporal scales for each of these processes. Furthermore, most of the variables traditionally used to report coral restoration success might be useful to describe organism, population and community levels of organization, and/or methods to raise and outplant corals. Yet, they often do not demonstrate “improvement” in ecosystem functioning or the recovery of key ecological or environmental processes that occur at different spatial and temporal scales across different habitats (Table 1).

**Table 1:** List of response variables used in coral restoration programs, advantages, disadvantages and recommendations.

Approach	Target	Metrics	Advantages	Disadvantages
Univariate	Colony/population	Outplants number/unit area	Good proxies of outplanting effort/capacity to breed corals	Only useful if the aim is to restore a coral population. Do not show improvement on population specific properties (e.g., size, sex population structure, reductive output). Not linked to ecosystem function
	Colony/population	Survivorship	Good proxy of methods of outplanting/performance of a colony	
	Colony/population	Growth rate	Good proxy of colony/genotype performance	
	Physical structure	Rugosity	Easy to compute with outstanding advances of photogrammetry	Multivariate attributes of structural complexity shrinked and underestimated
	Habitat	Live coral cover	Easy to estimate and interpret/compare	Does not provide information about community emergent properties. Live coral cover may remain univariant, despite of dramatic changes in species composition
		Associated fauna biomass/density	Easy to estimate and interpret/compare	Visual census is often focus on species thriving in the water column, but not necessarily the ones using the benthic habitat. Difficult to independently assign each counting to each of the experimental units (e.g., plots). Fish biomass/density may remain univariant, despite of dramatic changes in species composition associated to the benthic habitats. Does not necessarily provide information on ecosystem function
	Community	Similarity indices	Easy to compute and interpret in terms of percentage of similarity/dissimilarity	Visualization of patterns depends on how well-represented samples are in an ordination space. Some indexes are sensitive to joint absences
	Community	Alpha diversity indices	Species diversity is an emergent property of communities, indices are easy to compute. They offer information linking benthic community structure with associated communities	Not directly comparable, highly influenced by sampling effort, area and/or data collection approach. Often underestimate rare species
	Community	Beta diversity indices	More informative to understand ecological processes (e.g., succession) relevant for species diversity (e.g., turnover and variation)	Not easy to compute, multiple indices, not always straightforward to interpret
Multivariate	Community	Live coral cover per species	Easy to estimate and interpret/compare	Does not provide information linking coral community, with habitat structure and other associated species. Underestimate the importance of early colonizers in the ecological succession
	Community	Benthic cover	Incorporates/acknowledges a multivariate framework which is the essential problem that ecological restoration attempts to achieve	Taxonomic resolution to species level might not be equal for all benthic groups
	Community	Fish biomass/density per species or guilds	Incorporates/acknowledges a multivariate framework which is the essential problem that ecological restoration attempts to achieve	Difficult to link positive impacts of restoration activities with highly variable fish communities. No information showing how species are interacting (e.g., predation rates)
	Ecosystem	Reef sound scape	Relates soundscapes with ecosystem status using multivariate indices (e.g., aquatic complexity index, sound pressure level and acoustic enrichment)	Limited spatial and temporal scales to detect sounds specific to the restoration site. More research to find the mechanistic links between sound and ecosystem function.
	Ecosystem	Carbonate budgets	Incorporates/acknowledge the importance of a key multivariate process for coral reefs. Relatively easy to estimate from accretion and bioerosion rates.	Habitat dependent. Highly variable across habitats and highly dynamic environmental conditions.
	Ecosystem	Predation/consumption rates (e.g., Squidpop) Functional indices	Incorporates the importance of species interactions. Easy to standardize/estimate/compare across habitats. Accurate comparisons among ecosystems based on species traits.	Biased by the type/size/availability of baits. Oversimplification of food web and species interactions. Functional indices require intensive sampling to capture wide range of species traits. Not easy to interpret.
	Ecosystem	Economic valuation	Acknowledges the importance of linking ecosystem function with the human dimension.	Comparisons across sites depend on methods used as they might be subjective and sometimes based on perceptions and intuition.

### Common variables used to measure coral restoration success

There is a myriad of variables that can be used to measure coral restoration success [59]. From an experimental ecology point of view, all metrics chosen must be goal-based; or better yet, hypothesis-driven. In this section we describe the pros and cons of variables commonly used by coral restoration practitioners to report the outcome of their projects and we suggest alternatives to complement these metrics.

#### Number of outplants, survivorship, and growth rates.

The number of outplants and their survivorship and growth rates are almost universally used as indicators of coral restoration success [60–63] (Table 1). While these indicators are highly valuable for assessing improvement of population descriptive parameters (e.g., growth rate, mean, mode, skewness and kurtosis) and the success of propagation methods and technologies, they do not necessarily demonstrate successful restoration, rehabilitation, enhancement and/or replacement at community or ecosystem levels of organization (Table 1). Even if the scope of the effort is to benefit communities or ecosystems through outplant survival and growth, these indicators should be complemented by others that demonstrate if the habitats are sustaining biodiversity (e.g., foundational, benthic invertebrates, and fishes) and ecological succession (e.g., promotion of settlement of early colonizers) as well as promoting ecosystem function (e.g., species interactions) (Table 1). If the scope of the program is to test technologies, a different experimental framework that allows comparisons of method effectiveness and/or efficiency must be incorporated, whereby the methods and technologies become the treatments that must be compared in the laboratory and/or in the field against appropriate controls and procedural controls when necessary. Such tests should not constitute a full-fledged ecosystem restoration effort, nor should they be reported as such, unless they prove recovery/improvement of coral ecosystem functions. Finally, if coral farming is the focus of a program, practitioners should use a clear experimental framework aimed at increasing coral production, with minimal coral extraction, maximum effective return (i.e., outplants that survive to become reproductive) and reduced costs.

In addition, outplanting corals in a degraded habitat might alter the natural course of ecological succession and any new habitat created by the intervention might be far from sustaining the target community. Under natural circumstances, a coral climax community is reached 9–12 years after a disturbance [64], although such time series are rare in coral restoration [9]. Thus, monitoring early colonizers and/or cryptic species (e.g., foraminifera, hydrozoans, bryozoans, serpulid worms, encrusting crustaceans, predatory snails and echinoids [65,66]) would be extremely informative to better weight the success of interventions (Table 1). However, these taxa are difficult to sample, and their taxonomy is complex, therefore, the use of artificial recruitment monitoring system (ARMS) [67,68] or any artificial substrate could be combined with environmental DNA/metabarcoding techniques to characterize early successional changes in species composition before and after interventions are implemented. Unfortunately, eDNA and other methods designed to look at small species and early colonizers are still relatively expensive, expertise-intensive, and lack appropriate reference libraries, which might difficult the adoption of this approach by many restoration projects.

Ultimately, the experimental framework proposed here (and others, like that by Chapman in 1998 [51]) for coral restoration aims to describe long-term divergent (e.g., control vs. intervention treatments) and/or convergent (e.g., intervention vs. reference) trajectories of whole community structure and ecosystem function, rather than short-term comparisons of methods whose primary goal is to optimize the breeding and/or outplanting and survival of corals.

#### Live coral cover.

Increased live hard coral cover is commonly used as an indicator of restoration success (Table 1), likely because it is an essential ocean variable [69] and is generally regarded as the most direct indicator of coral reef "health" [70–72]. While coral cover could be used to assess restoration success, it is important to acknowledge several limitations. First, coral cover is provided by assemblages of scleractinian corals. Our current ability to grow corals in land-based and water-based nurseries is limited to only a few species [9,29,59], and of those, only a smaller proportion, particularly the *Acroporids* can grow fast enough to contribute with coral cover in intervention areas within 2–5 years. Thus, live cover could be used as an indicator of success if the target habitat is monospecific and/or has a limited number of species, or if the goal is to enhance, rehabilitate or even replace the intervention area rather than to restore it as it was. Furthermore, the extensive mortality of major reef builders due to diseases (e.g., SCTLD) and bleaching has produced dramatic declines in live coral cover [73]. Thus, estimating the number of colonies per unit area (i.e., density) could provide additional information to detect change, as coral populations have been depleted to low coral cover in many regions such as the Caribbean [74].

#### Abundance and biomass of associate fauna.

Fish counts have been a highly recommended metric to demonstrate coral restoration success [59]. Visual censuses conducted at a site-scale are traditionally used by practitioners to determine the density, size and biomass of fish associated with a section of the reef. From these surveys, total biomass and density are estimated to depict the abundance of fish per unit area using standard methods (e.g., AGRRA, Table 1). While fish counts provide relevant information for coral restoration projects, the approach to conducting fish surveys often comes with major caveats. First, many surveys have restricted species lists or focus on large and evident species, especially with volunteer divers, which by design cannot provide a holistic assessment of reef biodiversity. Second, many surveys ignore cryptobenthic species which are vital to reef functioning [75]. Many surveys also do not consider benthic invertebrates [16], even though these may have significant implications for the health and productivity of the reef and in some cases, live coral cover and restoration outcomes (e.g., crown-of-thorns sea stars, fireworms). Some monitoring programs, such as Reef Life Survey, look at all species, including cryptobenthic fish and invertebrates [76] and should be considered when devising a restoration monitoring strategy. Finally, fish and invertebrates constitute a direct link to key services arising from reefs, including herbivory, fisheries, and tourism. Thus, assessing faunal responses to restoration can not only reflect ecological progress but also the capacity of the restoration to sustain human well-being.

#### Structural complexity.

Reef physical structure largely defines its capacity to provide functionality, therefore, the recovery of structural complexity through coral outplanting is also taken as evidence of the reversal of ecosystem decline [77]. Recently, many restoration programs have adopted photogrammetry underwater techniques (e.g., Structure-from-Motion) to determine three-dimensional volume and rugosity as indicators of structural complexity. However, structural complexity is a multivariate property of the reef habitat (i.e., shape, size, volume, and diversity of different benthic organisms) and can be estimated at different spatial scales [2] and therefore, with different ecological interpretations. Even when rugosity has been highly useful to describe structure complexity declines in the Caribbean region [78], it is just one among many important variables that make the benthos heterogeneous [79,80] such as fractal dimension, topographic position, structural richness, and structural evenness [81]. Colony morphology in the form of volume should also be incorporated following a multivariate approach (Table 1).

### Indices to compare communities that can be used to measure restoration success

In community ecology, comparisons of species assemblages and/or biological communities in space (e.g., across environmental gradients) and through time (e.g., ecological succession) are extremely relevant as they can provide clues to key underlying processes that control the development, and importantly, the persistence of these assemblages [25]. While the multivariate approach provides more information about the structure of these assemblages or communities, the interpretation of multiple interacting variables (e.g., species relative abundances) might be daunting to most practitioners (Table 2). To overcome this problem, ecologists have developed metrics of distance and/or similarity that collapse the multivariate complexity into diversity indices aimed to represent samples in reduced ordination spaces [82]. These indices should be more informative to assess and/or report the outcomes of a restoration program dedicated to restoring coral communities and coral reef ecosystem function, recognizing that they are useful only when comparing within a system and pairs of samples. It is more difficult, however, to compare metrics of community dissimilarity across different efforts if, for instance, they do not share a common pool of species and/or are assessed differently (abundance vs. density).

**Table 2:** Common practices in coral restoration programs and recommendations for coral restoration projects aiming at supporting the recovery of community structure and ecosystem function.

Components of coral restoration experiment	Common practices	Recommended practice
Hypotheses	Hypothesis not included	Use hypothetical-deductive instead of Inductive/Baconian reasoning
	Hypothesis stated incorrectly	Include predictions (e.g., effect size), draft a clear null hypothesis for a restoration experiment
	Hypothesis confounded with objectives	Tailor SMART objectives to test your hypotheses
	Effect sizes and errors of inference are not set a priori	Stablish conservative effect sizes a priori and set low type I errors for statistical inference
	No clear idea of the target population	Include a clear conceptual framework to know in advance: (1) what is the level of organization to benefit and (2) what is the intention (restore, rehabilitate, enhance or replace)
Factorial structure	BACI and Restoration designs regarded as the same	Differentiate the purpose, predictions and hypotheses of BACI and Restoration designs
	Omission of reference sites	A reference is not a pristine condition. Check for options nearby the area to intervene and for historical data. Adapt to set objectives to improve, enhance or even replace
	Factorial design does not respond the hypothesis	Establish proper set of factors (fixed or random, orthogonal, nested) to test your hypothesis. Use controls and procedural controls if needed.
Replication, Randomization and assignment of treatments	Confusion of units of analysis: unreplicated treatments with multiple replicated samples	Incorporate conceptual framework of experimental design. Knowing the hypothesis helps to define the experimental units, knowing the target population helps to define the operation units.
	Pseudo replication	Avoid any form of pseudo replication as it might inflate type I error, a sensitive problem for restoration experiments
	No reference to randomization and interspacing of treatments	Make sure that response variables are independent between treatment, remove practitioner biases
Response variables	Confusion between metrics depicting aquaculture success, restoration efforts and ecosystem recovery	Separate metrics depicting restoration efforts from those showing recovery of ecosystem function
	Use of diversity indexes without acknowledging problems of sample size, species representativeness	Estimate sample coverage, use K and ABC curves to have a more mechanistic understanding of biodiversity
	Preference of univariate approach (e.g., coral cover, total biomass, rugosity)	Embrace multivariate approaches and use similarity indexes traditionally used to compare samples as a proxy of restoration success.
	Use of variables difficult to align with the conceptual framework of ecosystem functioning (e.g., ecological footprint)	Use proxies of functionality (e.g., predation rates, carbonate budgets, bioerosion, etc.) and include the human dimension (ecosystem goods and services)
Approaches for data analysis	Lack of identification of all sources of variation in the experiment and the analysis	Identify, understand and interpret in advance all sources of variation in the restoration experiment
	Lack of proper partition of the variance	Use statistical models that allow proper partition of variance, particularly interactions (e.g., ANOVA, MANOVA, GLM, GAM) and/or are more flexible to data assumptions (PERMANOVA).
	Use of non-parametric test unable to test for relevant interactions	The analysis of treatment x time interactions is the only statistical prove for restoration success and/or any other temporal trajectory recorded at control, intervention and reference sites

#### Similarity indices based on species abundance and presence/absence.

Biological assemblages can be compared in space and time using similarity/dissimilarity indexes [82,83]. For decades, many indices have been developed [84], however, the Bray-Curtis index of similarity and the Jaccard-Dice index are amongst the most used in ecological studies because of their simplicity to interpret and unique properties [82]. The Bray-Curtis index (S) is a standardized measurement of similarity between two objects (e.g., samples, sites) estimated from absolute differences in species abundance relative to the total abundance of species, expressed as a percentage (Table 1) [82]. The Jaccard-Dice index is similar, but calculations are made on species presence/absence rather than abundance [82]. Similarity indices such as the ones described in this section are often used to describe spatial and temporal changes in biological assemblages [82], and recently, species replacement or loss [85,86], which is essentially the goal of ecological restoration. However, sometimes a clear visualization of patterns depends on how well samples are represented in the ordination space and some of these indices are sensitive to joint absences (Table 2).

#### Diversity indices.

Diversity is an emergent property of communities and can be partitioned into Gamma (regional species pool), Alpha (local assemblage), and Beta (variation of species composition among assemblages) components [86]. Beta diversity may reflect two different phenomena, spatial species turnover and nestedness of assemblages, which result from two antithetic processes, namely species replacement and species loss, respectively [86]. Understanding the extent to which each of these processes drives community dissimilarity can help to more accurately interpret responses to restoration and predict subsequent consequences affecting ecosystem function, for example, by demonstrating which species are missing between the reference and restoration sites.

Indices of diversity are mathematical expressions that have been used for decades in community ecology [87], computed from estimations of species richness and the relative contribution of each species (evenness) in the community [82]. The major caveat of these indices is that all of them are highly influenced by the surveyed area because the number of species increases with sampling size [88]. In consequence, direct comparison of classic diversity indices (e.g., Shannon-Weiner) is only possible for cases with similar and/or “large” sampling efforts [88]. Even when samples are standardized by size, they will usually have different degrees of completeness, depending on the species abundance distributions in the communities being compared [88]. To overcome these limitations Cao and Lost (2012) developed new “coverage-based” rarefaction and extrapolation methods that allow fair comparison of samples across different assemblages and levels of effort [88]. Furthermore, richness omits the identity of species in the community, whereas diversity indices normally underestimate the abundance of rare species [89] (Table 1). Ecologists have proposed the use of rank-abundance (*k*) [87], abundance-biomass curves (ABC) [89], and diversity profiles [90], as analytic tools to better understand the underlying dynamic processes of species assemblages (e.g., niche partitioning). Thus, advanced methods to characterize diversity represent a better approach to comparing spatial and temporal changes in biodiversity before and after interventions (Table 2).

#### Functional diversity indices.

Functional diversity is a component of biodiversity that concerns what organisms do, rather than, for example, their taxonomic identity [91]. Thus, functional diversity is a measure of diversity that implicitly incorporates putative mechanisms of ecological interactions between species, and it provides a general approach for scaling from individual traits to properties of communities and ecosystems [91]. Specific descriptions of diversity indexes are revised elsewhere [91] and it is out of scope for this paper, however, they can be divided among five four categories: (1) measures that work on trait values directly (e.g., community weight means), (2) measures that work on distance matrices, (3) measures that work on functional dendrograms, (4) measures that concern multidimensional hypervolumes [92] including innovations for more representative estimation of the portion of the multivariate space being occupied by particular species [93] and (5) measures derived from bioenergetic models that quantify process-based ecosystem functions [94]. All these methods would rely on having species with clearly defined traits [95], although methods exist to impute or resolve unmeasured traits, as well as accommodate a mix of trait types (continuous vs. ordinal). A clear guidance for relevant decisions to choose among multiple functional traits is described elsewhere [96]. For our scope of analysis, the incorporation of functional diversity approaches would represent an important improvement for coral restoration science.

### Strategy for data analysis and interpretation

To test the success of restoration experiments, we must compare the trajectories of the chosen metrics in the control, reference, and intervention treatments before and after unique and/or multiple interventions take place. The workflow for data analysis is three-fold: (1) visualization of spatial and temporal patterns (i.e., exploration of the data), (2) formal test of null hypothesis and (3) interpretation of results [82].

### Visualization of spatial and temporal patterns

For univariate cases, there are many options to explore if there are spatial and temporal patterns in a dataset. However, in our context, whatever option that comparatively shows trajectories of the three levels of treatments (control, intervention and reference) as in Fig 3 is valid. For multivariate cases, ordination techniques are a better option (e.g., principal component analysis for environmental data and metric or non-metric multidimensional scaling for biological data). These techniques allow the visualization of community trajectories by using distance and/or similarity indexes [82].

### Formal test of null hypothesis

A key analytic step in statistical inference is to choose the best approach to test hypotheses. There are two important facts that must be considered to select the best choice to test if interventions to restore, rehabilitate, enhance or replace community structure and ecosystem function have been successful. First, as mentioned before, restoration requires complex designs with multiple sources of variation and interactions among factors in the experiment. Second, the data will unlikely satisfy classic assumptions of normally distributed errors, homogeneity of variance, and independence of errors. In consequence, the chosen methods should allow estimating robust effect sizes (e.g., through maximum-likelihood estimation), addressing non-normality (e.g., by specifying specific error distributions), and/or incorporating random variation for multiple sources of non-independence that arise from complex experiments (e.g., through fixed correlation structures or mixed-effects modeling).

Thus, for complex designs we strongly recommend semi-parametric tests based on permutations flexible to parametric assumptions (e.g., PERMANOVA) [97] and/or depending on the kind of dependent variable used (continuous or discrete), any family of Generalized Linear (Mixed-Effects) Models or even Generalized Additive Models in the presence of significant non-linearities, but only if the data meet the assumptions of these models [98,99].

### Interpretation of results

**Fig 4:**
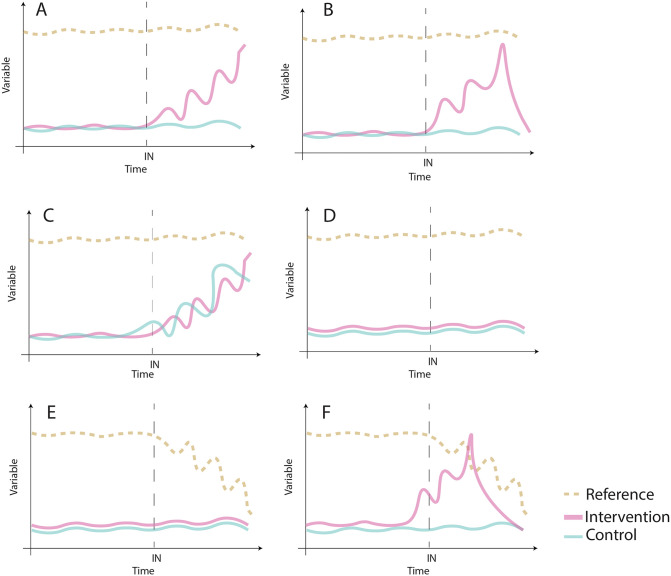
Examples of different hypothetical outcomes (treatment x time interactions) of a coral restoration experiment using a single variable and or an index (e.g., diversity, evenness, Bray Curtis similarity, rugosity). (a) successful restoration, (b) successful interventions not sustained in time, (c) successful interventions and natural recovery, (d) unsuccessful restoration, (e) unsuccessful restoration and decline of reference sites, (f) successful restoration not sustained in time with decline of both reference and intervention sites. Coral restoration experiments often entail multiple interventions (vertical blue bars) with potential cumulative long-term positive outcomes.

**Fig 5:**
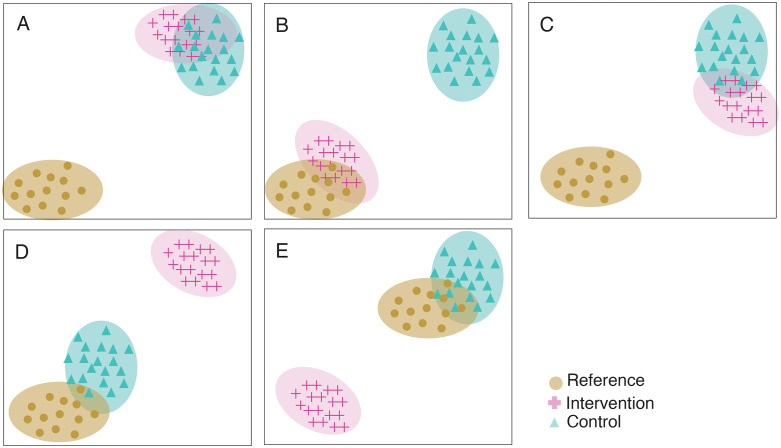
Different hypothetical non-Metric Multidimensional scaling (nMDS) ordinations in a Bray-Curtis ordination space depicting the possible outcomes of a coral restoration experiment using a multivariate approach. **(a)** Initial array before interventions, (b) successful restoration, (c) unsuccessful restoration, (d) unsuccessful restoration and natural recovery and (e) decline of reference sites.

Restoration success can be statistically proven by looking at meaningful factor interactions for the hypothesis being tested [51]. Particular attention must be paid to interactions between the treatments (i.e., control, reference and intervention) and time (before and after) as the experiment expects different temporal trajectories for these treatments [51] (Fig 4 a-e). However, it is important to look at other relevant interactions that could depict trajectories such as successful interventions not sustained in time because of the presence of disturbances leading to coral outplant mortalities (Fig 4b) and cases of natural recovery and trajectories recorded when multiple interventions are used during a project (Fig 4d). The same principle applies to coral restoration projects using a multivariate approach (Fig 5a-d). Using ordination techniques and multivariate tests, it is possible to represent relevant interactions to portray temporal trajectories of samples belonging to different treatments in a chosen ordination space/technique (e.g., MDS, nMDS, CCA).

In terms of scalability, if the design incorporates the three levels of treatments replicated across sites, localities and regions, all interactions involving time-x-treatment across these spatial scales must be properly interpreted to conclude that interventions were successful or not. For example, post-hoc contrasts of a significant interaction between time-x-treatment and site would allow practitioners to conclude whether interventions have been consistently successful at the scale of sites (e.g., hundreds of meters), or an interaction between time x treatment and locality-region would allow us to conclude if interventions have been consistently successful at the scale of localities (e.g., tens of kilometers) or regions (e.g., hundreds of kilometers). If interventions are consistently effective across various spatial scales, then interactions should not become statistically significant, and success can be inferred just by looking at the main effect of treatment and time.

## Analysis of current coral restoration efforts under the proposed experimental framework

In previous sections, we argue that coral restoration fits the definition of a long-term manipulative experiment [42,100], hence the importance of having a clear conceptual framework based on principles of ecological theory and experimental designs. This section analyzes specific examples of coral restoration studies conducted during the past 10 years to evaluate if these principles were used. Specifically, we reviewed five critical aspects: (1) clear hypothesis and predictions, (2) factorial structure of the coral restoration experiment, (3) proper replication, randomization and assignment of treatments, (4) variables used to conclude restoration success, and (5) approaches for data analysis (univariate and/or multivariate). In the next few years, we expect this framework will be adopted by restoration practitioners who have not envisioned their projects as we propose here.

### Hypothesis and predictions

Coral restoration guides published over the past five years acknowledge the importance of having hypotheses and predictions (often called SMART objectives) as part of the systematic design of coral restoration projects [29,101]. Furthermore, other authors acknowledged the importance of incorporating principles of experimental design into coral restoration projects [14,59]. However, in practice most coral restoration efforts do not apply these principles properly. This is particularly evident in cases where practitioners do not clearly state and make publicly accessible their projects’ goals to restore, rehabilitate, enhance, or replace the community structure and ecosystem function of reefs. For example, because coral restoration projects focus on outplanting a few breedable species (*in situ* and *ex situ*) across multiple habitats, replacement rather than restoration might be a common outcome. This frequently manifests in the Caribbean as outplanting *Acropora cervicornis* on relict *Orbicella* spp frameworks.

### Factorial structure of the coral restoration experiment

Often, coral restoration practitioners do not recognize restoration projects as long-term manipulative experiments, as terrestrial ecologists often do [102]. A common problem in coral restoration projects is the lack of control, reference and/or intervention levels of treatment [19,20,103,104] (Table 2). There are instances where the lack of reference sites is a logistical, rather than a conceptual, problem as finding “healthy” reefs seemed to be impossible [105]. In such cases, it is important to consider that a reference does not imply pristine conditions and, if data are available, sometimes the desired state could be a historical condition in a particular site or maintenance of a region-wide baseline. In cases where it is impossible to have a reference, practitioners must understand that with no references, it is impossible to know what is the desired community structure and ecosystem function the program aspires to reach with the interventions, and the only information that can be extracted from the experiment is a formal comparison between the control site (i.e., natural recovery) and the intervention site (i.e., recovery associated with the interventions). It is also impossible to understand whether any outcomes are attributable to the intervention or to broader regional patterns. However, we did find cases where the control, reference, and intervention treatments are included, but using a different terminology (e.g., degraded, negative control, restored and healthy reefs) [23,106,107] (Table 2). Another common problem in coral restoration experiments is the lack of procedural controls when needed (e.g., when predator exclusion devices are used or when a device is used to shade a coral nursery), and incorrect partition and/or examination of the variance accounted for all sources of variation in the experiment (e.g., controlled and uncontrolled random variability and covariates) (Table 2).

### Proper replication, randomization, and assignment of treatments

A common problem of coral restoration projects is the paucity of proper replication and misuse of the BACI experimental design (Table 2). For example, there is a paucity of quantification of the random effect of the experimental units (e.g., plots) nested within treatments (i.e., control, reference and intervention) and the spatial and temporal interactions derived from a restoration experiment [105]. In consequence, it is impossible to know if the outcome of interventions varies within sites for uncontrolled reasons, although it is up to the investigator to identify and gauge whether potential confounders exist and if their influence is sufficient to explain the observed outcomes [108]. Nonetheless, there are a few examples of proper factorial structure in coral restoration experiments [109,110].

Often, because of time and/or resource constraints, plots are only monitored once before and after interventions to conclude about the success of interventions [104]. This is a clear case of simple pseudoreplication as temporal and spatial patterns are confounded because time is not properly replicated [46,47]. Furthermore, normally coral restoration practitioners often confuse units of analysis by designing experiments with unreplicated treatments with multiple replicated samples and do not provide details of randomization/interspacing procedures utilized to assign treatments to experimental units and/or operation units to each treatment [104] (Table 2). Nonetheless, there are examples of restoration experiments with no pseudoreplication and proper assignment of treatments [109].

Coral restoration projects also often lack key mediators of restoration outcomes in the experimental framework. Given the spatial heterogeneity and the uniqueness of the local context where interventions occur, including covariates such as depth, existing coral cover, substrate and habitat type, and water chemistry can control for variation to provide a more direct and cleaner test of the success (or lack thereof) of the restoration treatment.

### Variables and approaches used to conclude restoration success

We found that coral practitioners prefer the univariate approach to report coral restoration success, whereas the multivariate approach is less used (Table 2). The most widespread strategy used by restoration practitioners is “coral gardening”, which involves harvesting coral fragments from naturally occurring stock colonies, and either transplanting fragments directly to a “benefited” area of reef (i.e., ecological footprint) or growing the fragments to a larger size in a nursery prior to transplantation [20,60–63,111]. These approaches typically focus on branching coral taxa that are fast-growing and easily fragmented [9,59,112]. Increased coral cover and fish biomass are the most common variables that restoration practitioners use to report success in their projects [112–115]. Coral restoration practitioners often use species richness, composition and alpha diversity as proxies of restoration success [114,116], however, beta diversity partition [85], dominance (*k*) and ABC curves are never used even though they are more informative and tied to ecological processes (Table 2).

There are examples of coral restoration projects that have used and/or acknowledged the importance of incorporating multivariate approaches to measure the outcome of restoration [19,117], but only one [118] specifically reported success using similarity indices designed to compare communities and species assemblages [119]. While different studies have used ordination techniques to show changes in benthic and community structure [105], none have statistically demonstrated nor interpreted the ecological meaning of an increase in similarity between intervention and reference sites and/or decrease in similarity between intervention and control sites as unequivocal evidence of restoration success. A detailed analysis of species identities that contribute with similarities/dissimilarities between treatments (control, reference and intervention) must be conducted for a clear interpretation of the spatial and temporal trends recorded before and after interventions.

Coral restoration practitioners are also recognizing the importance of incorporating concepts of ecological succession into their projects [65,105,115,117,118,120,121]. Coral restoration projects aimed at recovering communities and ecosystems seldom incorporate a functional approach [101,122] (Table 2). Coral restoration efforts focus on outplanting corals to create habitats that promote recovery and/or foster species interactions [123] but never estimate multiple ecosystem functions. There are increasing efforts to enhance key ecological processes such as herbivory to improve the outcomes of coral gardening [124–128]. Other low cost-methods, such as, “Squidpop,” can be used as a comparative proxy of ecosystem function (i.e., predation intensity) in coastal marine ecosystems [129,130], but have not yet been used to demonstrate recovery of functionality due to coral restoration, as they have in in seagrasses, mangroves and oyster reefs [131].

Another approach recently used in coral restoration is the analysis of acoustics as a function of the number of species present in a reef. For example, comparing the soundscapes of healthy, restored and degraded reefs by using manual counts of biotic sounds (i.e., phonic richness) [11] and two commonly used computational analyses: (1) the aquatic complexity index (ACI) and (2) the sound pressure level (SPL). Authors found no soundscape differences between the restored and healthy reefs, whereas the degraded reefs had different acoustic profiles. There are cases where acoustics is used as an intervention to enhance coral and fish larval recruitment (i.e., acoustic enrichment techniques) [132–134]. One limitation of this approach is the narrow spatial scales at which positive responses have been documented [133]. While acoustics is a promising field in coral restoration [135,136], there are still gaps in understanding how soundscapes are mechanistically linked with specific ecosystem emergent properties and the provision of services they provide to human societies.

Lastly, socioeconomic variables have been used as indicators of coral restoration success [20,112,137]. For example, restored reefs have been shown to recover their aesthetic as a direct and/or indirect consequence of coral restoration efforts [126]. There are also interesting applications of ecotourism to carry out restoration efforts, which in a roundabout way constitutes a service arising from the reef by supporting local economies while simultaneously promoting restoration [138].

## Conclusions

The advances of coral restoration in the past decade are indisputable, particularly for the unification of terminologies and the capacity to produce and outplant corals. Technological advances and research aimed at finding scalable technologies to scale up restoration will always be essential for coral restoration science to improve our capacity to produce and outplant corals as well as to reduce time, optimize cost of interventions and monitor the outcomes faster and more accurately. However, coral restoration practitioners do not always apply principles of experimentation properly, partly because the field-based practice of supplying more corals to the reef has often been separated from the academic science of evaluating the impacts of these interventions on reefs over time. This disconnect is dangerous because restoration outcomes could be misattributed to the intervention in the absence of a rigorous experimental framework as proposed by academics. We found there is a paucity of examples that utilize the establishment of hypotheses, an experiment design, and monitoring of ecosystem function-based variables distinguishing between restoration, rehabilitation, improvement and replacement while setting objectives, targets and tailoring interventions.

Coral restoration practitioners do not often explicitly state their predictions and null hypothesis nor establish expected effect sizes for their interventions *a priori* for their statistical inferences. These problems are derived from conceptual/philosophical clarity rather than correct application of specific terminologies. Experimental (e.g., plots, sites) and operation/evaluation units are often confounded by coral restoration practitioners. In most cases, samples/operation units are replicated, while treatments are not, and in consequence cases of pseudoreplication are common. Coral restoration is often based on Baconian (trial and error) philosophy, while hypothetical/deductive reasoning is less popular. The common practice of coral restoration often omits a reference site and/or confounds it with the control assuming this treatment plays the same role as in a BACI or Beyond BACI design. There are instances where the reference is omitted because of the lack of “pristine” or “healthier” reefs nearby intervention sites. However, adopting an experimental framework, particularly the monitoring of intervention, control, and reference sites, can address these issues and further provide enormous diagnostic insight, adaptive management potential, and empowerment to coral restoration practitioners, funders, and government agencies to support more understanding and successful use of restoration interventions in the future. It is in fact the only way that we can truly demonstrate a restoration project or intervention as “successful.”

Success is primarily reported with variables depicting the capacity to culture and/or outplant corals based on the implementation of various technologies. Univariate metrics of coral cover and survival are the most common indicators of success for practitioners that prefer the univariate approach. Multivariate assessments are less used and/or biased towards linking benthic community changes driven by coral outplanting and the fish communities established at intervention sites, rather than comparing a control or reference site. There are only a few examples where (dis)similarity indices are used to infer restoration success and fewer examples of hypothesis testing with proper partition of sources of variation and interpretation of ecological meaning of these sources, particularly those depicting treatment-x-time interactions among factors. More efforts to incorporate ecological therory such as concepts of ecological succession, emergent assemblages such as fishes and invertebrates, and functionality based on species traits and interactions to evaluate success are needed, particularly evaluating taxa that might be using coral outplants as a habitat during early successional stages. Finally, there is an urgent need to incorporate the human dimension in the form of ecosystem goods and services into coral restoration experiments.

We do acknowledge there are barriers to implementing our proposed framework, but they can be overcome. Technical expertise in experimental design and data analysis can be incorporated by fostering cooperation among practitioners. Projects can focus on enhancing colony survival or improving (not necessarily restoring) coral populations. For communities and ecosystems levels of organization, metrics are beyond more complex, and results will require long-term investments. Therefore, finding sustainable mechanisms for coral restoration projects is critical to improve the outcomes of coral restoration science. Despite the higher requirements, we believe this framework should be attractive to funders who seek assurances about the actual outcomes of their investments. Further, improved indicators can link other properties of the reef (e.g., percent hard coral cover) to those most relatable to human interests, such as tourism and fisheries.

While the paucity of a rigorous experimental framework in coral restoration has not precluded progress across the globe, implementing the recommendations above will keep improving the capacity for restoration practitioners and the community at large to assess success in the future, to better learn from failures, and to fairly compare and synthesize across restoration efforts at a time where restoration is at the forefront of global environmental policy. As coral restoration continues to scale, scientists, governments, practitioners and funders must embrace and invest in restoration experimental design, monitoring and evaluating systems that measure success based on ecological outcomes rather than activities inputs.

## Supporting information

S1 Supplementary Schematic representation of an experimental design for a coral restoration experiment. Three levels of treatments are represented in different colors: intervention, control and reference. Experimental units (plots) are boxes nested within treatments. Observations before and after interventions (doted boxes) repeated multiple times (t1 to tn). Dots in smaller boxes are outplants or any operation units from which variables are collected. Treatments are truly replicated in plots; observations are replicated within each plot and time is truly replicated.
(TIF)
