# Supplementary figures and images for: Towards a systematic framework to assess restoration success of interventions in coral reef ecosystems
Source: PLoS One. 2026 Mar 9;21(3):e0331083. doi: 10.1371/journal.pone.0331083 (PMC12970865; doi:10.1371/journal.pone.0331083)

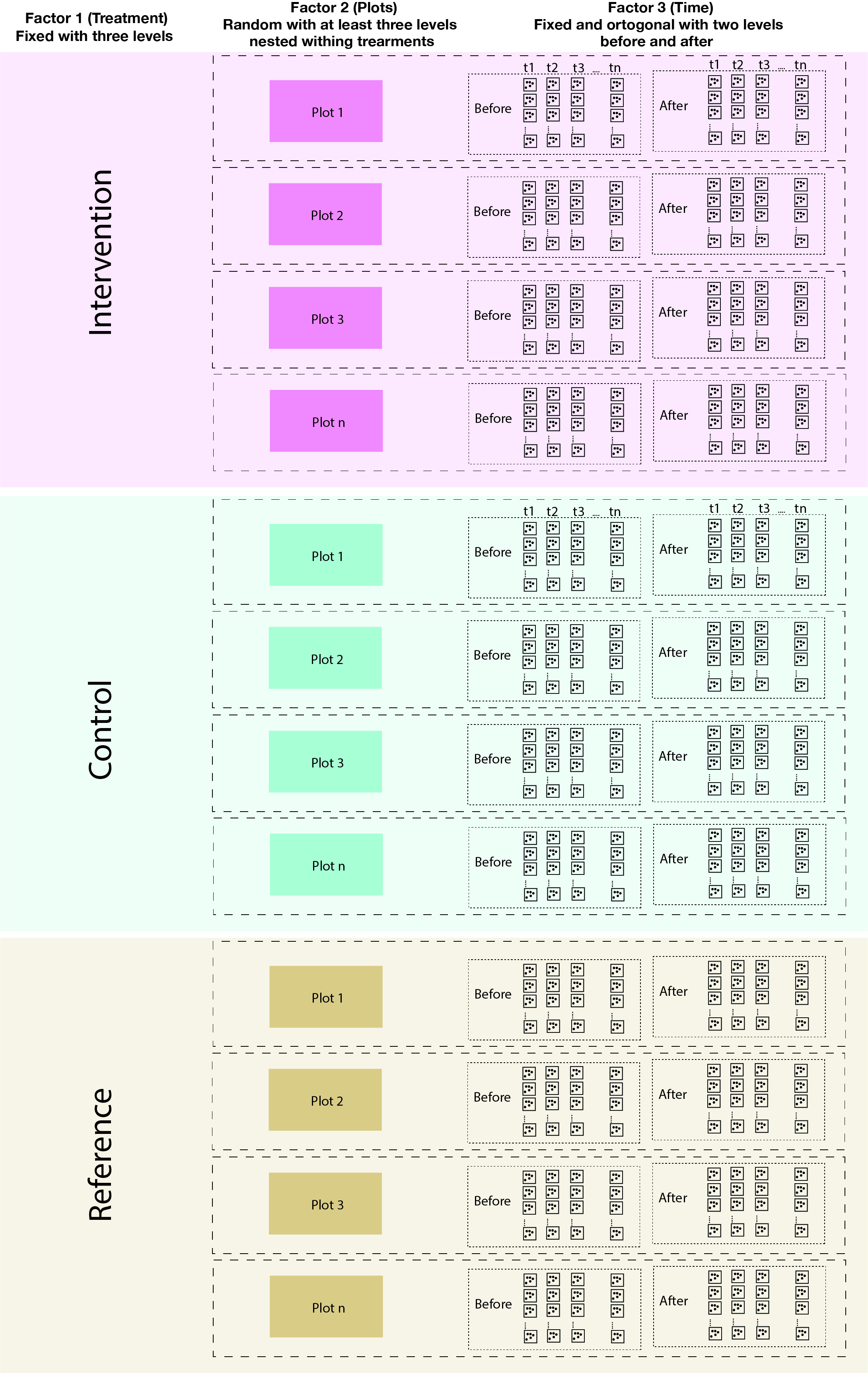

Supplement: S1 Supplementary — Three levels of treatments are represented in different colors: intervention, control and reference. Experimental units (plots) are boxes nested within treatments. Observations before and after interventions (doted boxes) repeated multiple times (t1 to tn). Dots in smaller boxes are outplants or any operation units from which variables are collected. Treatments are truly replicated in plots; observations are replicated within each plot and time is truly replicated. (TIF) [file pone.0331083.s001.tif]
